# Researchers’ Intuitions About Power in Psychological Research

**DOI:** 10.1177/0956797616647519

**Published:** 2016-07-20

**Authors:** Marjan Bakker, Chris H. J. Hartgerink, Jelte M. Wicherts, Han L. J. van der Maas

**Affiliations:** 1 Department of Methodology and Statistics, Tilburg School of Social and Behavioral Sciences, Tilburg University; 2 Department of Psychology, Psychological Methods, University of Amsterdam

**Keywords:** power, survey, methodology, sample size, effect size, open data, open materials

## Abstract

Many psychology studies are statistically underpowered. In part, this may be because many researchers rely on intuition, rules of thumb, and prior practice (along with practical considerations) to determine the number of subjects to test. In Study 1, we surveyed 291 published research psychologists and found large discrepancies between their reports of their preferred amount of power and the actual power of their studies (calculated from their reported typical cell size, typical effect size, and acceptable alpha). Furthermore, in Study 2, 89% of the 214 respondents overestimated the power of specific research designs with a small expected effect size, and 95% underestimated the sample size needed to obtain .80 power for detecting a small effect. Neither researchers’ experience nor their knowledge predicted the bias in their self-reported power intuitions. Because many respondents reported that they based their sample sizes on rules of thumb or common practice in the field, we recommend that researchers conduct and report formal power analyses for their studies.

Despite the existence of alternative analytical techniques (Rouder, Speckman, Sun, & Morey, 2009; Wagenmakers, Wetzels, Borsboom, & van der Maas, 2011), and notwithstanding criticism (e.g., Nickerson, 2000), null-hypothesis significance testing (NHST) remains the main statistical tool in the analysis of psychological research data (Bakker & Wicherts, 2011; Nuijten, Hartgerink, van Assen, Epskamp, & Wicherts, 2015; Wetzels et al., 2011). Much recent debate on how researchers use NHST in practice has concerned the inflation of the number of Type I errors, or rejection of the null hypothesis when it is in fact true (Asendorpf et al., 2013; Bakker, van Dijk, & Wicherts, 2012; Simmons, Nelson, & Simonsohn, 2011; Wagenmakers et al., 2011). Reducing the possibility of Type II errors is another important consideration in improving the quality of studies, however: Studies should be well powered (Fiedler, Kutzner, & Krueger, 2012; Simmons et al., 2011).

It has long been argued that researchers should conduct formal power analyses before starting data collection (Cohen, 1965, 1990), yet it continues to be the case that many studies in the psychological literature are statistically underpowered (Bakker et al., 2012; Cohen, 1990; Maxwell, 2004). Specifically, given the typical effect sizes (ESs) and sample sizes reported in the psychological literature, the statistical power of a typical two-group between-subjects design has been estimated to be less than .50 (Cohen, 1990) or even .35 (Bakker et al., 2012). These low power estimates appear to contradict the finding that more than 90% of published studies in the literature have *p* values below the typical threshold for significance (i.e., α = .05; Fanelli, 2010; Sterling, Rosenbaum, & Weinkam, 1995). This apparent discrepancy is often attributed to the combination of publication bias (i.e., the nonreporting of nonsignificant results; Rosenthal, 1979) and the use of questionable research practices in the collection and analysis of data (John, Loewenstein, & Prelec, 2012; Simmons et al., 2011). Despite the centrality of power in NHST (Gigerenzer, 2004), formal power analyses are rarely reported in the literature. Sedlmeier and Gigerenzer (1989) found that none of the 54 articles published in the 1984 volume of the *Journal of Abnormal Psychology* reported the power of the statistical tests that were presented. In a more recent and fairly representative sample of 271 psychological articles that involved the use of NHST (Bakker & Wicherts, 2011), only 3% of the authors explicitly discussed power as a consideration in designing their studies. Thus, it appears that sample-size decisions are hardly ever based on formal and explicitly reported (a priori) power considerations.

Here, we consider another explanation of the common failure to conduct sufficiently powerful studies, namely, researchers’ intuitions about statistical power. In a classic study, Tversky and Kahneman (1971) showed that even quantitatively oriented psychologists underestimated the randomness in small samples. In addition, when Greenwald (1975) asked social psychologists what the acceptable Type II error rate was, the average response was around .27, which means that an acceptable level of power would be .73, which again is markedly higher than the overall power estimates for published studies, as reported by Cohen (1990) and Bakker et al. (2012). These results suggest that researchers may intuitively overestimate the power associated with their own research and that of others (i.e., in their role as reviewers).

Given the centrality of power in the debate regarding reproducibility and replicability of research in psychology and beyond (e.g., Asendorpf et al., 2013; Button et al., 2013; Gilbert, King, Pettigrew, & Wilson, 2016; Open Science Collaboration, 2015), we surveyed psychology researchers on their practices, intuitions, and goals related to statistical power. In our first study, respondents assumed the role of either researcher (reporting on their own studies) or reviewer (assessing their peers’ studies) in answering questions about typical and acceptable cell sizes (*n*s), ESs, power levels, and alpha levels. In addition, respondents in the researcher condition indicated how they typically determined their sample size in planning studies, and those in the reviewer condition indicated how they assessed sample sizes in other researchers’ studies. This survey informed us about the typical study from the viewpoints of both researchers and reviewers. In our second study, respondents estimated the actual power of several research designs and the sample size that would be required to achieve a power of .80 in various research designs.

## Study 1

### Method

#### Subjects

We collected all e-mail addresses of the corresponding authors of the 1,304 articles published in 2012 in *Cognitive, Affective, & Behavioral Neuroscience; Cognitive Psychology; Developmental Psychology; European Journal of Work and Organizational Psychology; Health Psychology; Journal of Consulting and Clinical Psychology; Journal of Experimental Social Psychology; Personality and Individual Differences; Psychological Methods;* and *Psychological Science*. After removing 80 duplicate e-mail addresses and 5 physical addresses, we invited 1,219 researchers from various subdisciplines in psychology to participate in our online survey on the Qualtrics Web site in September 2013. Eighty-four e-mails bounced; thus, we assume that we were able to contact 1,135 researchers. We expected this sample to be sufficiently large for the (mostly descriptive) analyses that we had planned.

Of all the contacted researchers, 499 (44%) started the survey. Note that respondents would have been counted twice in this number if they started the survey, did not complete it, and then started it a second time after we sent a reminder. We could not send a personalized reminder because we did not want to be able to connect contact information with the responses given. Seven respondents who started the survey chose not to give informed consent and therefore did not complete it. A total of 291 (26%) respondents finished the survey. Respondents were randomly assigned to complete the reviewer’s version or the researcher’s version of the questionnaire. One hundred sixty-nine respondents completed the latter version, and 122 respondents completed the former version. We focus our discussion on the results obtained in analyses including only those respondents with complete data, except as noted.

#### Survey

We developed two versions of a short survey containing 10 questions (available at Open Science Framework, https://osf.io/5t0b7/). The first version contained questions to be answered from a researcher’s perspective, and the second version contained questions to be answered from a reviewer’s perspective. The last 3 questions (concerning respondents’ research field, statistical knowledge, and number of publications) were the same for the two versions. Results for 8 of the questions are discussed in this article, and results for the other 2 are presented in the Supplemental Material available online. Specifically, here we discuss the respondents’ descriptions of how they generally determined their sample size (researcher condition only, because answers to the corresponding question in the reviewer condition were hard to classify) and their assessments of the acceptable Type I error rate, the power level regarded as satisfactory, the cell size typically considered sufficient, and the typically expected ES (in Cohen’s *d*) for an independent-samples *t* test. The design did not involve any additional dependent or independent variables.

### Results

#### Deciding on sample size

A total of 197 respondents answered the open question on determination of sample size from a researcher’s perspective (note that for this analysis, we included answers from respondents who did not finish the survey). Two independent raters scored whether the answers could be assigned to one or more of five different categories. The raters agreed in 93% of the cases (Cohen’s κ = .80). A power analysis was mentioned by 93 (47%) of the respondents (although 20 of these respondents, or 22%, also mentioned practical constraints, such as available time and money). Overall, 40 respondents (20%) stated that practical constraints determined their sample size. Furthermore, 45 respondents (23%) mentioned some rule of thumb (e.g., 20 subjects per condition), 41 respondents (21%) based sample sizes on the common practice in their field of research, and 18 respondents (9%) wanted as many subjects as possible, to have the highest possible power to detect an effect.

#### The typical study

Because responses in the researcher condition were very similar to those in the reviewer condition, we present results for the two conditions combined (see the Supplemental Material for results separated by condition). As the distributions were not normal and included outliers (histograms and medians are presented in the Supplemental Material), we report the trimmed means (*M_t_*s; 20% trimming) and used robust statistics to increase power and to protect against an incorrect estimation of the Type I error rate (Bakker & Wicherts, 2014; Welch, 1938; Wilcox, 2012; Yuen, 1974).

The average expected ES was 0.39, which is somewhat lower than the estimated mean ES obtained in large-scale meta-analyses of psychological research (*d* = 0.5 on average; Anderson, Lindsay, & Bushman, 1999; Lipsey & Wilson, 1993; Meyer et al., 2001; Richard, Bond, & Stokes-Zoota, 2003; Tett, Meyer, & Roese, 1994). However, these meta-analyses probably overestimated the mean ES because of the publication bias often present in meta-analyses (Bakker et al., 2012). Note that the average expected ES found in Study 1 is comparable to the (original) mean ES (*d* = 0.402) of 100 studies in psychology that were recently subjected to replication (Open Science Collaboration, 2015). The average acceptable cell size reported by our respondents was 34.6, which is somewhat higher than previous estimates of mean cell sizes based on the published literature (20–24 subjects; Marszalek, Barber, Kohlhart, & Holmes, 2011; Wetzels et al., 2011). The average reported acceptable levels for α and power were .05 and .80, respectively. Responses to these questions, in particular, seemed to reflect a common standard, as 83% of our respondents reported that the acceptable α level is .05, and 69% reported that power of .80 is sufficient.

In answer to our question about determining sample size, one respondent indicated, “I usually aim for 20–25 subjects per cell of the experimental design, which is typically what it takes to detect a medium effect size with .80 probability.” However, for an independent-samples *t* test with 20 to 25 subjects in each condition and *d* of 0.5 (medium ES), the actual power lies between .34 and .41, which is approximately half the power that the respondent mentioned. Considering that 53% of the respondents in the researcher condition indicated that they did *not* generally conduct power analyses and 23% reported using some rule of thumb, we wondered whether respondents’ intuitive power analyses were accurate. To investigate this, we calculated the power of a study with α, ES, and cell size equal to the trimmed means obtained in Study 1, using the pwr package in R (Champely, 2009). Such a study would have power of .35. (When we calculated power separately for each respondent’s reported values of α, ES, and *n*, we found that the trimmed mean power across respondents was .40.) We also calculated the required cell size given the trimmed means for α, ES, and power, and found that it would be 105 subjects, which is 3 times as many subjects as respondents’ trimmed mean for *n*.

A robust within-subjects Yuen *t* test (Wilcox, 2012; Yuen, 1974) indicated that respondents’ reported acceptable power levels differed significantly from the calculated power based on their responses to the other questions, *t*(171) = 19.38, *p* < .001, ξ = .82, 95% confidence interval (CI) for the difference = [.36, .44]. We also calculated the bias for each respondent individually (calculated power – reported power). The trimmed mean bias was –.34; 80% of the respondents showed a negative bias (calculated power lower than desired power), and 33% showed a negative bias with an absolute value larger than .5.

## Study 2

A majority of the respondents in Study 1 reported that power of .80 is satisfactory, and this is the common standard advised by Cohen (1965) and other researchers. Hence, it might be that our respondents gave the normative answer even though they knew that it was not in accordance with the other values they reported. The goal of Study 2 was to measure researchers’ power intuitions more directly, by asking them to estimate the power of research designs with α, ES, and *N* specified. Additionally, we presented examples of research designs with α and ES specified, and asked respondents to estimate the number of subjects needed to reach a power of .80.

### Method

#### Subjects

We collected all e-mail addresses of the corresponding authors of articles published in 2014 in the same journals as used in Study 1. After removing 1 duplicate e-mail address and 1 e-mail address from a lab member familiar with the hypotheses, we invited 1,625 researchers to participate in our online survey on “statistical intuitions” in February 2015. We did not conduct a formal power analysis because we considered this sample sufficiently large for the purposes of estimation.

Of all the contacted researchers, 404 (24.9%) started the survey, and 214 (53.0%) of those who started the survey completed it. Respondents were randomly assigned to one of three sample-size versions of the survey. Sixty-seven respondents completed the small-*N* version (*N* = 40), 81 completed the medium-*N* version (*N* = 80), and 66 completed the large-*N* version (*N* = 160). We report the results obtained when our analyses included only the respondents with complete data.

#### Survey

Our short survey contained 10 questions (available at Open Science Framework, https://osf.io/5t0b7/). The first 3 asked the respondents to estimate the power of independent-samples (two-tailed) *t* tests in three research situations, which differed in the ES (Cohen’s *d* = 0.20, 0.50, or 0.80); α was set at .05 throughout. Depending on the condition to which respondents were assigned, the total *N* specified was 40, 80, or 160. In the next 3 questions, we asked the respondents to estimate the sample sizes required for an independent-samples *t* test to have a power of .80 given expected ESs (Cohen’s *d*) of 0.20, 0.50, and 0.80; each ES was accompanied by the corresponding correlation (.10, .24, or .37, respectively), and α was again set at .05. Next, we tested respondents’ understanding of what power is with a single multiple-choice question. Finally, we asked them to indicate how often they conducted a power analysis (7-point Likert scale), to assess their own statistical knowledge (10-point scale), and to indicate their main subfield of psychological research. The design did not involve any additional dependent or independent variables.

### Results

#### Intuitions about power and sample size

**Fig. 1.**
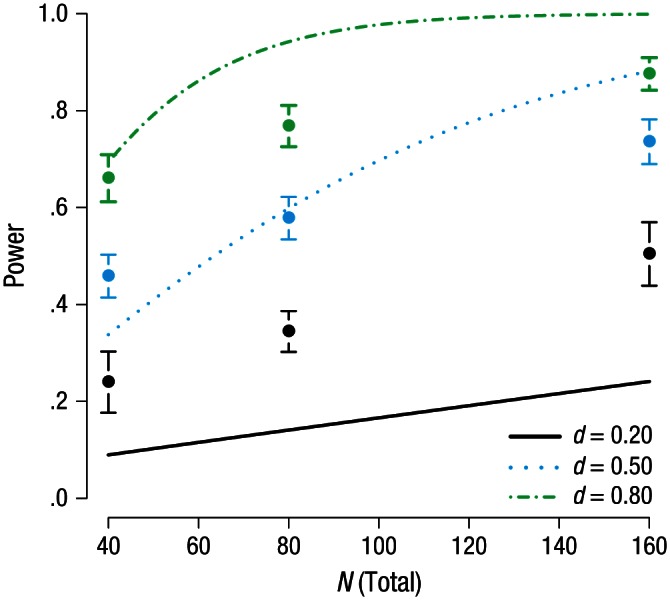
Results from Study 2: respondents’ mean estimate (20% trimmed mean) of the power of the presented research design for each combination of sample size and expected effect size. The error bars represent 95% confidence intervals, and the lines indicate the true power of studies with the three expected effect sizes as a function of total sample size.

We calculated the true power of the research designs presented to the respondents using the pwr package in R (Champely, 2009); these values are presented in Table 1 and Figure 1, along with the 20% trimmed means and 95% CIs for the respondents’ estimates. Most respondents were not able to estimate the true power values well. The true power lay within the 95% CI for only one scenario in the medium-*N* condition (when *d* = 0.50) and one scenario in the small-*N* condition (when *d* = 0.80). The vast majority of respondents (89%) overestimated power for the small-ES scenario. This is especially worrisome given that small ESs are typically found in psychological research (Open Science Collaboration, 2015) and were reported as typical by respondents in Study 1. When the ES was large and *N* was greater than 80, respondents underestimated the power of the *t* test in the design we presented to them.

**Table 1.** Results From Study 2: Respondents’ Estimates of Power and the True Power for the Research Designs

*N*	*d* = 0.20 (small ES)		*d* = 0.50 (medium ES)		*d* = 0.80 (large ES)	
	True power	Estimated power	True power	Estimated power	True power	Estimated power
40	.09	.240 [.177, .303]	.34	.459 [.414, .503]	.69	.660 [.612, .709]
80	.14	.344 [.302, .386]	.60	.578 [.534, .622]	.94	.768 [.726, .811]
160	.24	.504 [.439, .570]	.88	.736 [.690, .782]	> .99	.876 [.842, .909]

Note: The table presents the 20% trimmed means of the power estimates, with 95% confidence intervals inside brackets. ES = effect size.

A comparable pattern was found when respondents estimated the sample sizes required to obtain a power of .80 in an independent-samples *t* test, given a specific expected ES. Table 2 gives the true sample sizes needed in these cases along with respondents’ estimates (20% trimmed means). When the expected ES was large, respondents overestimated the required sample size by about 25 subjects, on average. Respondents’ mean estimate was quite close to the actual value when the expected ES was medium. When the expected ES was small, however, the required sample size was underestimated by 95% of the respondents. Whereas respondents estimated, on average, that 216 subjects were needed, 788 subjects would actually be needed to obtain sufficient power in the case of such a small effect. Given that respondents in Study 1 indicated that their typical ES was around 0.4, on average, our results suggest that researchers typically underestimate the sample sizes needed for studying effects that they deem to be typical. Unexpectedly, we did find a difference in sample-size estimates among the three conditions: Respondents in the large-*N* condition gave the highest estimates. This might be a carryover effect from the questions asking respondents to estimate the power of research designs (e.g., effect of anchoring and adjustment; Epley & Gilovich, 2006).

**Table 2.** Results From Study 2: Respondents’ Estimates of the Required Sample Size and the True Required Sample Size to Reach a Power of .8

Required sample size	*d* = 0.20 (small ES)	*d* = 0.50 (medium ES)	*d* = 0.80 (large ES)
True	788	128	52
Estimated	216 [196, 236]	124 [114, 134]	77 [72, 83]

Note: The table presents the 20% trimmed means of the sample-size estimates, with 95% confidence intervals inside brackets. ES = effect size.

#### Other factors

To explore possible influences on respondents’ power intuitions, we looked at the data from both studies. We focused especially on the small-ES situations, because these are common in psychology and also because respondent’s intuitions were the least accurate for these situations. First, we found that respondents who reported doing power analyses to determine their sample sizes did not estimate power better than those who did not report conducting power analyses. Almost half of the respondents in the researcher condition in Study 1 indicated that they generally used a power analysis to determine their sample size (although they might not conduct a power analysis for every single study). The average calculated power for this group of respondents (*M_t_* = .46, 95% CI = [.37, .55]) was not significantly higher than that for the remaining respondents in the researcher condition (*M_t_* = .42, 95% CI = [.34, .51]). Furthermore, the amount of bias did not differ significantly between respondents who mentioned typically doing power analyses (*M_t_* = −.31, 95% CI = [−.40, −.22]) and those who did not (*M_t_* = −.30, 95% CI = [−.39, −.22]).

Next, for Study 2, we used a principal components analysis to summarize respondents’ answers to the questions regarding their understanding of what power means (question correctly answered by 168 respondents, or 78.5%), how often they conducted power analyses, and how good their statistical knowledge was. The first component explained 50% of the variance, and we used hierarchical regression analyses to investigate whether scores on this component predicted estimates of power and required sample sizes. (Separate results for the three questions, including full regression tables, are available in the Supplemental Material.) The dependent variables were the power and sample-size estimates for each presented research design. In the first model for each dependent variable, we included only component score as a predictor; in the second model, we added condition; and in the third model, we added the interaction between component score and condition. We report results based on Model 2, which was always selected on the basis of the change in *R*^2^ except for predicting sample size in the small-ES scenario, in which case none of the models fitted the data. No interactions between condition and component score were found. We did not find a significant effect of component score on power estimates for the small-ES scenario (*b* = −0.01, *t* = −0.98, *p* = .329). However, when the ES was medium or large, respondents with higher component scores had higher (and hence more accurate) power estimates (*b* = 0.02, *t* = 2.26, *p* = .025, and *b* = 0.04, *t* = 3.94, *p* < .001, respectively). Furthermore, when the specified ES was large, respondents with higher component scores gave smaller estimates of the sample size required to achieve a power of .80 (*b* = −12.89, *t* = −2.56, *p* = .011), which again resulted in estimates closer to the true value. Component score did not significantly predict sample-size estimates when the ES was small or medium (*b* = 16.54, *t* = 1.33, *p* = .185, and *b* = −6.30, *t* = −1.05, *p* = .296, respectively).

In Study 1, respondents’ self-reported statistical knowledge correlated with neither calculated power nor bias. In addition, robust regression analyses revealed that number of publications did not significantly predict either calculated power or bias.

Finally, we did not find any significant differences between research fields in Study 1 respondents’ calculated power (full results are presented in the Supplemental Material). For Study 2, we combined cognitive psychology and neuroscience, and added the 2 respondents from forensic psychology to the “other” category, because of the small number of respondents in these categories. With a robust 3 (condition) × 9 (research field) two-way analysis of variance using the trimmed means, we tested for differences between research fields in power and sample-size estimates. We found that the research fields differed only in sample-size estimates for the situation in which the ES was small (*F* = 41.43, *p* = .006). These estimates were lowest for respondents from the fields of cognitive psychology and neuroscience and highest for respondents from personality and developmental psychology. However, the highest mean sample-size estimate (by the respondents from personality psychology) was 276, which is still far removed from the true required sample size of 788 in that scenario.

## Discussion

It has long been noted that the statistical power of studies in the psychological literature is typically too low (Bakker et al., 2012; Cohen, 1990; Maxwell, 2004). The results of the current studies, involving more than 500 psychology researchers, offer insight into why this may be so. Specifically, for studies of effects expected to have the most typical magnitude, respondents overestimated power and consequently underestimated the required sample size. When asked about how they normally determined sample sizes in their own studies, more than half of our respondents indicated that they did not use a power analysis, which may explain why such analyses are presented in fewer than 3% of psychological articles (Bakker & Wicherts, 2011). Much research in psychology appears to be planned without formal power analysis, and many researchers appear to use rather intuitive approaches in determining their sample sizes.

In our first study, the calculated power based on respondents’ reported acceptable sample sizes and expected ESs was only half of the power respondents indicated they wanted to achieve. The power intuitions of more than 75% of respondents resulted in calculated power that was lower than desired. Results were similar for respondents who answered as researchers and those who answered as reviewers. In our second study, 89% of respondents overestimated the power of studies with small expected ESs, and 95% underestimated the sample size required for sufficient power when the ES was small. When the expected ES was small, the true sample size needed to reach a power of .80 was more than 3 times the respondents’ mean estimate of the required sample size. This is worrisome, as the results of our first study and replication studies show that ESs are often quite small in psychology (Open Science Collaboration, 2015). In combination with publication bias, the (strategic) use of small sample sizes and research designs that are underpowered results in inflated Type I error rates, biased ES estimates, distorted meta-analytical results, and nonreplicable findings (Bakker et al., 2012; Open Science Collaboration, 2015).

Even researchers who stated that they typically used formal power analyses had poor power intuitions. In line with earlier work showing the same poor statistical intuitions among general and mathematical psychologists (Tversky & Kahneman, 1971), our studies indicate that greater self-reported statistical knowledge and experience are not related to better power intuitions in the most common cases (when the ES is small). Only when the underlying ES was large did we see some apparent advantage of knowledge and experience. In our second study, we found a small difference between research fields in the estimates of required sample sizes when the ES was small. However, the true required sample size is more than 2.5 times the mean estimate of respondents from the research field with the highest sample-size estimate (personality psychology).

We focused on a between-subjects experimental design because it is a common and basic design in psychology. Nevertheless, it is possible that some of our respondents were more familiar with other research designs that have different associations between sample size and power (e.g., within-subjects designs are typically more powerful). However, if experience with research designs had influenced our results, power intuitions should have differed more between subfields that typically use different research designs. Future research could focus on power intuitions related to other research designs, such as within-subjects and correlational designs. We also found some evidence for carryover effects. However, the questions calling for power estimates and the questions calling for sample-size estimates showed the same pattern of results: large discrepancies between estimated and actual values in all conditions when the ES was small. Furthermore, the response rate in both studies was quite low (26% and 13%, respectively), and researchers who are knowledgeable about power are probably overrepresented in this sample because of their interest in the subject. Therefore, we expect that a more balanced sample would show even larger overestimation of power and underestimation of required sample sizes in research designs.

Poor intuitions about power may lead to incorrect inferences concerning nonsignificant results. Researchers often conduct multiple small (and therefore likely underpowered) studies of the same underlying phenomenon (Francis, 2014; Hartgerink, Wicherts, & van Assen, 2015). Given the flawed power intuitions we observed, it is quite likely that researchers dismiss nonsignificant outcomes in such studies as due to methodological flaws (i.e., “failed studies”) or feel inclined to interpret nonsignificant outcomes as reflecting a true null effect, although in fact these outcomes might be false negatives (Hartgerink et al., 2015; Maxwell, Lau, & Howard, 2015). These small (often exploratory rather than confirmatory; Wagenmakers, Wetzels, Borsboom, van der Maas, & Kievit, 2012) studies should be combined within a meta-analysis to estimate an underlying mean effect (and confidence interval) and to ascertain whether there is heterogeneity in the underlying ES (Bakker et al., 2012).

Our results lead us to the following recommendations for using NHST. First, researchers should always conduct a formal power analysis when planning a study (preferably, such an analysis would be part of an institutional review board’s approval or part of preregistration of the study), and they should report this power analysis in their manuscript, together with a description of their sample. This will force researchers to explicate their sample-size decisions and will likely lead to better-powered studies. Second, considering that often no appropriate ES estimation is available and that our results indicate that intuitions for exponential power functions are often suboptimal and potentially linear, we recommend that power analyses be accompanied by inspection of the implications of a range of ES estimates, especially at the lower end of this range. This will help researchers understand the exponential relations involved in statistical power and the considerable impact of seemingly small changes in ES estimates (see also Perugini, Gallucci, & Costantini, 2014). Third, reviewers should check whether indeed a formal power analysis has been conducted (Asendorpf et al., 2013) and whether it is sound. Fourth, confirmatory studies, or core studies in a research line, should be sufficiently powerful and preregistered (Asendorpf et al., 2013; Wagenmakers et al., 2012). If researchers conduct exploratory studies or analyses, these should be presented as such and possibly combined in a meta-analysis to provide estimates of the mean effect and possible heterogeneity of effects (Bakker et al., 2012).

In the current climate of debate about replicability, reproducibility, and reporting standards, researchers and reviewers should collaborate in assessing the reliability of research results (Asendorpf et al., 2013). Both parties may misestimate the power of studies, regardless of their self-assessed statistical expertise. There is really only one way to improve studies: power them up.

## Supplementary Material

Supplementary material

Supplementary material
