# Supplementary material for: Researchers’ Intuitions About Power in Psychological Research
Source: Psychol Sci. 2016 Jul 20;27(8):1069–77. doi: 10.1177/0956797616647519 (PMC4976648; doi:10.1177/0956797616647519)
Supplement: Supplementary material [file suppl-material.pdf]

DOI: 10.1177/0956797616647519

Supplementary Online Material

Study 1

Invitation letter

Dear dr. ...,

We contact you because you published in Psychological Science in 2012. We would be very grateful if you would complete a short survey that pertains to common research practices in your field. This survey consists of 10 short questions and will take no more than 3 minutes of your time.

To participate in the survey, please click on the following link:
Take the Survey<link>

All results will be analyzed in aggregate only and answers will never be associated with individual participants.

We would greatly appreciate your participation.

Marjan Bakker (m.bakker1@uva.nl), and Jelte M. Wicherts (j.m.wicherts@uvt.nl)

Reminder prompt

Dear dr. …,

This is a quick reminder to complete our survey on research practices. This survey consists of 10 short questions and will take approximately 3 minutes of your time.

To participate in the survey, please click on the following link:

All results will be analyzed in aggregate only and answers will never be associated with individual participants.

If you have already completed the survey, thank you very much for doing this and please accept our apologies for sending this reminder.

Best regards,

Marjan Bakker (m.bakker1@uva.nl), and
Jelte M. Wicherts (j.m.wicherts@uvt.nl)

DOI: 10.1177/0956797616647519

Trimmed means, medians, and distribution of the different variables

Table S1 reports the trimmed means (20%) and the medians of the different variables presented in Table 1 for the participants in the researcher and reviewer condition, separately. The distributions of the variables are given in Figure S1. The unjust removal of outliers can increase the Type I error rate, but keeping real outliers in your data and non-normal distributions can decrease the power of parametric tests. Furthermore, deciding on outliers is often very subjective and these choices might suffer from p-hacking or biases. Therefore, we choose to use robust statistics, which have high power when assumptions are violated (see for an extensive review of this issue Bakker & Wicherts, 2014). We report trimmed means (20%) and use the Yuen Welch test to compare two independent groups.

Table S1

Trimmed means (medians) of typical (for researchers) and desired (for reviewers) Alpha, Effect Size, *N*, and power given by the respondents, and the power estimates and bias.

	Researchers	Reviewers
α	.05 (.05)	.05 (.05)
Effect size (*d*)	0.40 (.40)	0.36 (.35)
N (cell size)	37.1 (35)	32.2 (30)
Reported power	0.80 (0.80)	0.79 (0.80)
Calculated power (overall)	0.41 (0.38)	0.30 (0.27)
Calculated power (based on individual answers)	0.44 (0.44)	0.34 (0.34)
Bias	-0.31 (-0.32)	-0.39 (-0.39)

Notes: The calculated power (overall) was based on these trimmed means for ES and *N*. The individual power calculations were based on *N*, ES, and α given by individual respondents. The bias was calculated as: (calculated power (individual) – reported power).

DOI: 10.1177/0956797616647519

Figure S1: distributions

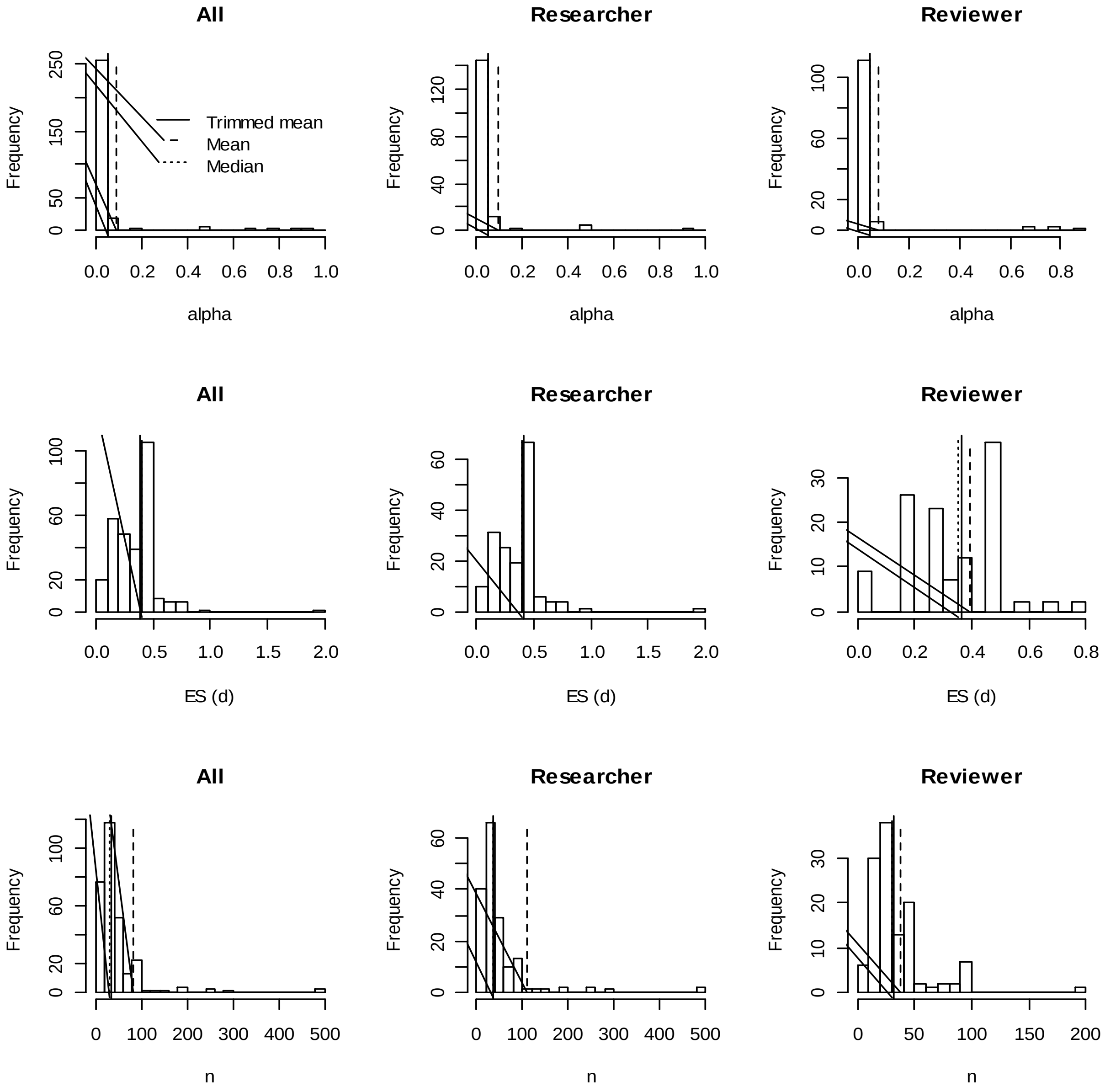

Notes: The typical ES and *N* as given by the participants contained some extreme outliers that disturbed the plots. Therefore, we show only the histograms of responses within the range 0 and 3 (for *d*) and 0 and 501 (for *N*). Not included in the plot are therefore: one participant (researcher condition) with *d* = 99999, one participant (reviewer condition) with *d* = 5, and one participant (researcher condition) with *N* = 10000.

DOI: 10.1177/0956797616647519

Figure S1 (continued)

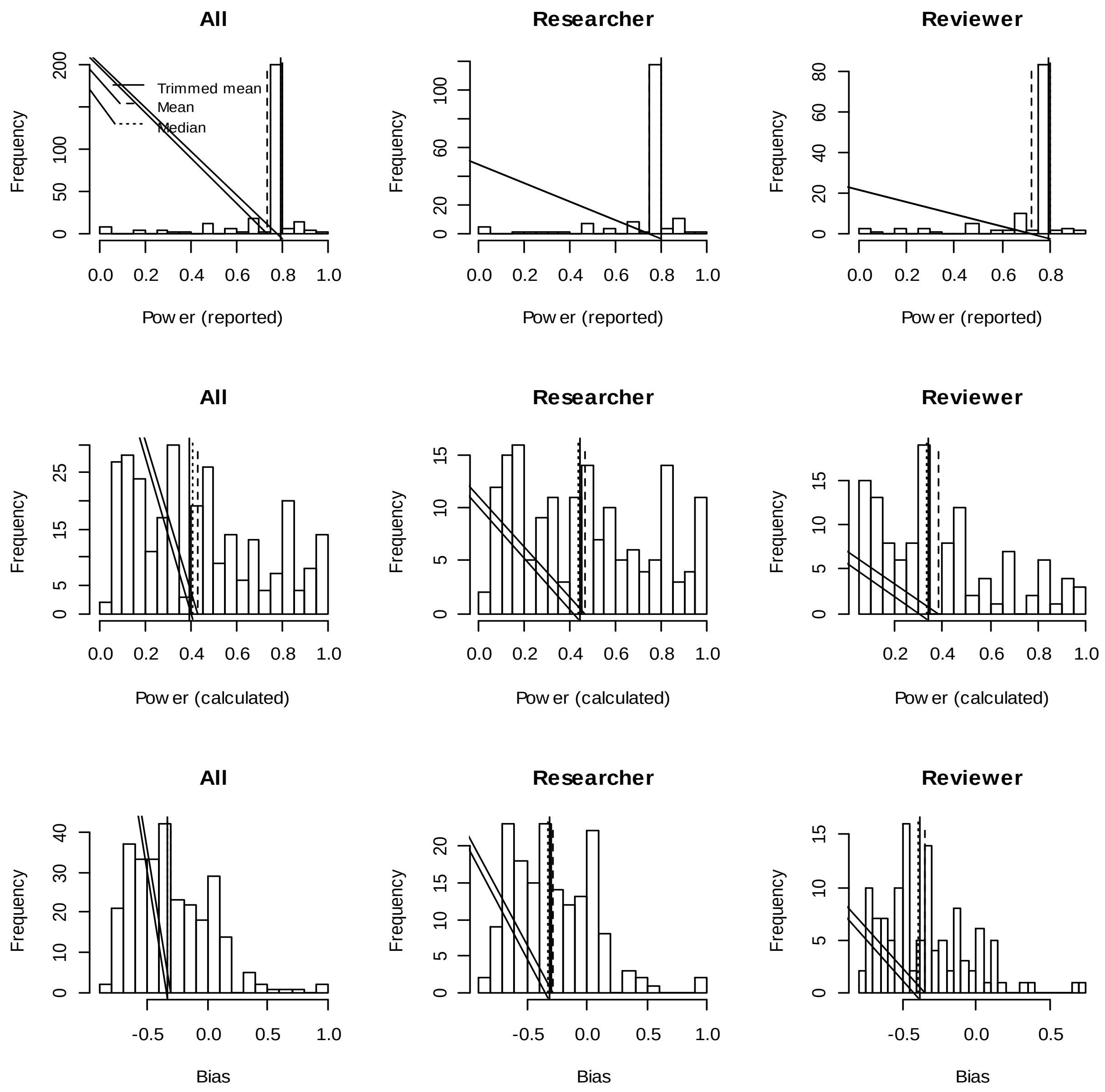

DOI: 10.1177/0956797616647519

Differences between researcher and reviewers

We did not find significant differences in typical α, ES, N and reported power between the researcher and reviewer conditions. The typical α had no variance (after trimming) and therefore the Yuen-test could not be applied; ES: $t_y(159.2) = 1.73$, $p = .086$, $\xi = 0.15$, 95% CI = [-.01, .09]; N: $t_y(175.0) = 1.77$, $p = .078$, $\xi = 0.18$, 95% CI = [-.56, 10.41]; power: $t_y(73) = 1.46$, $p = .15$, $\xi = 0.10$, 95% CI = [-.00, .02]. The trimmed mean of the individually calculated power was somewhat larger for the researchers (95% CI = [.38, .50]) as opposed to the reviewers (95% CI = [0.28, .40]). The trimmed mean of the bias was somewhat less for the researchers (95% CI = [-.37, -.24]) as opposed to the reviewers [-.45, -.33].

Statistical knowledge

To see whether respondent's self-assessed statistical knowledge was related to better power intuitions, we correlated (Spearman's Rank Order correlation was used because of non-normality) the calculated power and bias with respondent's self-reported statistical knowledge. In both conditions, we failed to find significant correlations (Power, Researcher condition: $r_s = -0.01$, $p = .865$; Power, Reviewer condition: $r_s = .03$, $p = .763$; Bias, Researcher condition: $r_s = -0.11$, $p = .144$; Bias, Reviewer condition: $r_s = -0.10$, $p = .265$).

Table S2
Number of participants (%) in each number of publication category and the trimmed mean of the calculated power and bias per category.

Number of publications	N	M_t Power	M_t Bias
< 5	41 (14%)	0.35	-0.38
5-15	69 (24%)	0.36	-0.33
16-30	83 (29%)	0.40	-0.35
31-50	39 (13%)	0.42	-0.36
51-100	31 (11%)	0.51	-0.25
> 100	28 (10%)	0.44	-0.35

Number of publications

We also investigated whether the number of publications of respondents was related to their power intuitions. In Table S2 the trimmed means of the calculated power and bias are presented for the different publication categories. A robust regression, by using the rlm() function of the MASS package in R, with condition and number of publication as predictors failed to show a significant main effect of number of publications or an interaction between the research output and condition. The number of publications or the interaction between number of publications and condition failed to significantly

DOI: 10.1177/0956797616647519

predict α, ES, and power. However, the number of publications positively predicted *N* (b = 3.58, p = .002).

Table S3
Number of participants per research field and for each condition separately, and the trimmed means of statistical knowledge, recalculated power, and bias per research field.

	N	*N* Researcher	*N* Reviewer	M_t Statistical Knowledge	M_t Power	M_t Bias
Clinical	43 (15%)	19 (11%)	24 (20%)	6.7	.47	-.31
Cognitive	29 (10%)	13 (8%)	16 (13%)	6.3	.34	-.38
Developmental	42 (14%)	28 (17%)	14 (11%)	7.3	.40	-.35
Forensic	2 (1%)	1 (1%)	1 (1%)	6.5	.37	-.03
Health	12 (4%)	6 (4%)	6 (5%)	7.0	.47	-.31
Industrial organizational	19 (7%)	11 (7%)	8 (7%)	6.8	.31	-.45
Neuroscience	14 (5%)	10 (6%)	4 (3%)	6.6	.43	-.31
Personality	39 (13%)	22 (13%)	17 (14%)	7.3	.37	-.32
Quantitative	10 (3%)	6 (4%)	4 (3%)	8.5	.44	-.38
Social	81 (28%)	53 (31%)	28 (23%)	6.5	.40	-.33
Total	291	169	122			

Research Field

We investigated possible differences in power intuitions between the different research fields. In Table S3 the trimmed mean of the calculated power and bias are presented for each research field separately. Because only two participants indicated Forensic psychology as their main field of research, we could not include them in a two way ANOVA of trimmed means. We used the function t2way() of the WRS package, which does not give the *df* or ES. Furthermore, we find slightly different results for the main effects of condition compared with the results of the robust *t* test that we used before, because the means are trimmed in every cell (9*2) and for the *t* test in only two cells. We did not find a main effect of research field (F_t = 7.32, p = .622) or an interaction between field and condition in estimated power (F_t = 15.26, p = .155). Similarly, bias showed neither a main effect for research field (F_t = 3.01, p = .951), nor an interaction between field and condition (F_t = 7.69, p = .580).

We found no differences between sub-fields or interactions with condition for the reported α and reported power (both no variance after trimming). For ES we did not find a main effect of sub-field (F_t = 16.95, p = .113), but did find a significant interaction effect with condition (F_t = 23.18, p = .032). The estimated ES differed between the conditions for participants whose main field of research was Health Psychology, Personality Psychology, and Social Psychology with an estimated ES for participants in the researcher condition of M_t = 0.29, M_t = 0.40, and M_t = 0.41, respectively, and for the participants in the reviewer condition of M_t = 0.46, M_t = 0.27, and M_t = 0.30, respectively. We also found a main effect of sub-field on *N* (F_t = 21.44, p = .032), but no interaction effect with condition (F_t = 17.31, p = .081).

DOI: 10.1177/0956797616647519

Especially, the trimmed mean of participants from Clinical Psychology (M_t = 44.6) or Personality Psychology (M_t = 47.1) showed higher values of the reported *N* than participants from Cognitive Psychology (M_t = 27.1) or Neuroscience (M_t = 26.3).

DOI: 10.1177/0956797616647519

Additional questions

In Study 1 we included a question about whether respondents would prefer to conduct (or see in manuscript as reviewer) multiple small studies or rather one large study. We found that differences between the conditions in whether respondents would prefer 5 studies (*N* = 20), 4 studies (*N* = 25), 2 studies (*N* = 50) or 1 study (*N* =100; see Table S4). A 2 (researcher v. researcher) by 4 (number of studies) χ^2 test was significant ($\chi^2(3) = 23.3$, $p < .001$, $\varphi = .28$). A majority of the participants who answered the question from a researcher's perspective preferred one large study, whereas most participants who answered the question from a reviewer's perspective preferred two smaller studies.

Table S4
Number of researchers (%) that preferred 5 studies (*N* = 20), 4 studies (*N* = 25), 2 studies (*N* = 50) or 1 study (*N* = 100) per condition.

	Researchers	Reviewers
5 studies (*N* = 20)	8 (5%)	9 (7%)
4 studies (*N* = 25)	11 (7%)	9 (7%)
2 studies (*N* = 50)	46 (27%)	63 (52%)
1 study (*N* = 100)	104 (62%)	41 (34%)
Total	169	122

In Study 1 we also included a question about outliers. In each condition we had 2 versions. In one version removing the outliers would change the results from nonsignificant to significant and in the other version, both with and without outliers the results were significant and not substantially different. We asked the respondents whether they would report the results with the outlier, the results without the outlier, both the results, or other. The results are presented in Table S5. We see a strong preference for reporting both results.

Table S5
Number of researchers (%) that preferred studies (*N* = 20), 4 studies (*N* = 25), 2 studies (*N* = 50) or 1 study (*N* = 100) per condition.

	Researchers		Reviewers	
	Different results	Same results	Different results	Same results
With outliers	3 (3%)	15 (19%)	1 (1%)	10 (18%)
Without outliers	5 (6%)	11 (14%)	5 (7%)	4 (7%)
Both	76 (84%)	51 (65%)	58 (87%)	38 (69%)
Other	6 (7%)	2 (3%)	3 (4%)	3 (5%)
Total	90	79	67	55

DOI: 10.1177/0956797616647519

Study 2

Invitation letter

Dear fellow psychological scientist,

We contact you because you published an article in a high-impact journal in 2014. We would be very grateful if you would complete a short survey that pertains to statistical intuitions in research. This survey consists of 10 short questions and will take no more than 3 minutes of your time.

To participate in the survey, please click on the following link:

${l://SurveyLink?d=Take the Survey}

Or copy and paste the URL below into your internet browser:

${l://SurveyURL}

All results will be analyzed in aggregate only and answers will never be associated with individual participants. We disabled any form of IP-address logs on Qualtrics.

We would greatly appreciate your participation.

Marjan Bakker (m.bakker_1@uvt.nl),

Jelte M. Wicherts (j.m.wicherts@uvt.nl), and

Chris H.J. Hartgerink (c.h.j.hartgerink@uvt.nl)

Reminder prompt

Dear fellow psychological scientist,

This is a quick reminder for our request to take part in our survey on statistical intuitions. This survey consists of 10 short questions and will take approximately 3 minutes of your time.

To participate in the survey, please click on the following link:

${l://SurveyLink?d=Take the Survey}

Or copy and paste the URL below into your internet browser:

${l://SurveyURL}

All results will be analyzed in aggregate only and answers will never be associated with individual participants. We disabled any form of IP-address logging on Qualtrics.

If you have already completed the survey, thank you very much for doing this and please accept our apologies for sending this reminder.

Best regards,

Marjan Bakker (m.bakker_1@uvt.nl),

DOI: 10.1177/0956797616647519

Jelte M. Wicherts (j.m.wicherts@uvt.nl), and

Chris H.J. Hartgerink (c.h.j.hartgerink@uvt.nl)

Additional descriptives

Table S6 and S7 contain the average power and sample size estimates based on all available data. We see the same patterns as presented in the paper.

Table S6
True power and the 20% trimmed means [95% confidence intervals] of the power estimates given by the participants in the different research situations.

		d = 0.20 (small)	*d* = 0.50 (medium)	*d* = 0.80 (large)
N = 40	True power	0.09	0.34	0.69
	Estimated power	0.242 [0.188,0.296]	0.463 [0.422,0.504]	0.658 [0.612,0.703]
N = 80	True power	0.14	0.60	0.94
	Estimated power	0.338 [0.297,0.378]	0.574 [0.533,0.616]	0.763 [0.719,0.806]
N = 160	True power	0.24	0.88	>.99
	Estimated power	0.493 [0.429,0.557]	0.719 [0.669,0.768]	0.846 [0.794,0.897]

Table S7
True sample sizes to reach a power of .8 and the 20% trimmed means [95% confidence intervals] of the sample size estimates given by the participants for an independent *t*-test and different underlying effect sizes.

	d = 0.20 (small)	*d* = 0.50 (medium)	*d* = 0.80 (large)
True sample size	788	128	52
Estimated sample size (20% trimmed mean)	215 [196,234]	123 [113,133]	77 [71,83]

Other factors

To investigate the influence of other factors we summarized the three questions (actual knowledge of power, how often the respondent conducted power analyses, and a self-assessment of statistical knowledge) by means of a PCA. The first component explained 50% of the variance and we used the component scores (CS) to investigate whether these scores predicted estimates of power and sample sizes. We used hierarchical regression analyses. In the first model, we included only CS, in the second model we added condition by means of two dummy coded variables where sample size condition small serves as the reference category (D1 = 1 when sample size condition is medium; D2 = 1 when sample size condition is large), and in the third model we added the interaction between CS and the dummy coded condition. Table S8 presents the results of the hierarchical regression analysis with power estimates as the dependent variable for small, medium, and large underlying ES, separately. Table S9 reports these results with sample size estimations as the dependent variable. We selected the best fitting model (bold text) based on the R^2 change. With power estimates as dependent variable differences between the conditions are expected, we therefore focus on the effect of CS. For all three underlying ES, model 2 fitted best, and only when the ES were medium or large did respondents with a

DOI: 10.1177/0956797616647519

high CS have higher (and hence more accurate) power estimates (*b* = 0.021 and *b* = 0.041, respectively). With sample size as dependent variable, we did not expect any differences between the conditions. Nevertheless, we observed an effect of condition when the underlying ES are medium or large. These are probably carry-over effects. Furthermore, when the underlying ES was large, respondents provided smaller sample size estimates (*b* = -12.89), again resulting in estimates being closer to the true value.

To investigate the two questions with a continuous scale separately we ran the same hierarchical regression analyses with how often the respondent conducted power analyses (PA) as predictor (Table S10 and S11) and self-assessment of statistical knowledge (SK) as predictor (Table S12 and S13). With PA as a predictor and power estimates as dependent variable, Model 2 was selected for all three underlying ESs. The predictor PA was only significantly higher (and hence more accurate) when the underlying ES was medium (*b* = 0.016) or large (*b* = 0.020). With sample size as dependent variable, we expected no difference between the conditions. Nevertheless, we did observe the same pattern as with CS, with an effect of condition when the underlying ES was medium or large. Again, these results are probably due to carry-over effects. Furthermore, when the underlying ES were large, respondents had smaller sample size estimates (*b* = -12.67), again resulting in estimates closer to the true value. When underlying ES is small, Model 3 seems to fit significantly better, than Model 2. However, overall, Model 3 was not statistically significant ($F(5,208) = 1.873$, $p = 0.100$).

With SK as a predictor and power estimates as dependent variable, Model 2 was selected for all underlying ESs. The predictor SK was only significantly higher (and hence more accurate) when underlying the ES was large (*b* = 0.022). With sample size as dependent variable, we again witnessed the same pattern as with CS, with an effect of condition when the underlying ES was medium or large. SK is not a significant predictor for any of the underlying ESs.

To investigate the influence of actual knowledge of power, which is dichotomously scored, on power and sample size estimates, we used a robust 3 (condition) by 2 (correct/incorrect) ANOVA for trimmed means. With a small ES, we found an interaction between condition and answering the question on the power estimate ($F_t = 9.350$, $p = .027$), which makes it difficult to interpret any differences between participants who answered the question correctly and those who answered the question incorrectly. We also found this question to predict the sample size when ES was small ($F_t = 4.721$, $p = .035$). Participants who answered the question correctly showed higher estimates of the sample size, which were closer to the true value. When ES was medium we found an interaction between condition and answering the question correctly on estimating sample size ($F_t = 10.450$, $p = .012$). Participants in the small and medium sample sizes conditions who answered the power question incorrectly had a somewhat higher sample size estimate than participants who answered the power question correctly. For participants in the large sample size condition, this relation was the other way around.

DOI: 10.1177/0956797616647519

Table S8
Hierarchical regression analysis results with power estimates as dependent variable and component score (CS), condition and their interaction as predictors.

	Model 1			Model 2			Model 3		
	Coef	(SE)	p	Coef	(SE)	p	Coef	(SE)	p
Small									
ΔR^2	0.012		0.107	**0.168**		**0**	0.01		0.264
CS	-0.019	0.012	0.107	**-0.011**	**0.011**	**0.329**	-0.021	0.021	0.316
D1				**0.066**	**0.032**	**0.041**	0.064	0.032	0.05
D2				**0.217**	**0.034**	**0**	0.212	0.034	0
CS*D1							0.034	0.028	0.226
CS*D2							-0.006	0.028	0.824
Medium									
ΔR^2	0.006		0.28	**0.261**		**0**	0.02		0.057
CS	0.012	0.011	0.28	**0.021**	**0.009**	**0.025**	-0.015	0.018	0.404
D1				**0.102**	**0.028**	**0**	0.096	0.028	0.001
D2				**0.252**	**0.029**	**0**	0.249	0.029	0
CS*D1							0.052	0.024	0.028
CS*D2							0.049	0.024	0.042
Large									
ΔR^2	0.04		0.003	**0.149**		**0**	0.015		0.145
CS	0.034	0.011	0.003	**0.041**	**0.01**	**0**	0.009	0.02	0.638
D1				**0.089**	**0.031**	**0.004**	0.083	0.031	0.007
D2				**0.2**	**0.032**	**0**	0.197	0.032	0
CS*D1							0.051	0.026	0.055
CS*D2							0.038	0.026	0.155

Table S9
Hierarchical regression analysis results with sample estimates as dependent variable and component score (CS), condition and their interaction as predictors.

	Model 1			Model 2			Model 3		
	Coef	(SE)	p	Coef	(SE)	p	Coef	(SE)	p
Small									
ΔR^2	0.008		0.185	0.011		0.323	0.009		0.377
CS	16.537	12.429	0.185	18.535	12.509	0.14	-9.593	23.69	0.686
D1				-7.509	36.623	0.838	-12.203	36.788	0.74
D2				45.143	38.693	0.245	43.292	38.847	0.266
CS*D1							38.042	31.404	0.227
CS*D2							40.009	31.698	0.208
Medium									
ΔR^2	0.008		0.18	**0.03**		**0.038**	0.003		0.745
CS	-8.115	6.037	0.18	**-6.299**	**6.014**	**0.296**	-6.855	11.427	0.549
D1				**8.286**	**17.608**	**0.638**	8.411	17.745	0.636
D2				**44.747**	**18.603**	**0.017**	45.846	18.739	0.015
CS*D1							-4.556	15.148	0.764
CS*D2							6.33	15.29	0.679
Large									
ΔR^2	0.038		0.004	**0.041**		**0.01**	0.003		0.711
CS	-14.709	5.081	0.004	**-12.895**	**5.03**	**0.011**	-6.191	9.556	0.518
D1				**10.051**	**14.728**	**0.496**	11.19	14.839	0.452
D2				**45.146**	**15.56**	**0.004**	45.694	15.669	0.004

DOI: 10.1177/0956797616647519

CS*D1							-9.568	12.667	0.451
CS*D2							-9.012	12.786	0.482

Table S10
Hierarchical regression analysis results with power estimates as dependent variable and power analysis (PA), condition and their interaction as predictors.

	Model 1			Model 2			Model 3		
	Coef	(SE)	p	Coef	(SE)	p	Coef	(SE)	p
Small									
ΔR^2	0.003		0.428	**0.175**		**0**	0.003		0.657
PA	-0.007	0.009	0.428	**-0.005**	**0.008**	**0.535**	0.002	0.016	0.9
D1				**0.067**	**0.032**	**0.04**	0.08	0.089	0.37
D2				**0.22**	**0.034**	**0**	0.292	0.094	0.002
PA*D1							-0.003	0.021	0.878
PA*D2							-0.018	0.022	0.411
Medium									
ΔR^2	0.013		0.097	**0.254**		**0**	0.005		0.461
PA	0.014	0.008	0.097	**0.016**	**0.007**	**0.024**	0.003	0.014	0.848
D1				**0.103**	**0.028**	**0**	0.045	0.077	0.562
D2				**0.247**	**0.029**	**0**	0.154	0.08	0.056
PA*D1							0.014	0.018	0.424
PA*D2							0.023	0.019	0.215
Large									
ΔR^2	0.021		0.035	**0.134**		**0**	0.004		0.617
PA	0.018	0.009	0.035	**0.02**	**0.008**	**0.012**	0.01	0.016	0.543
D1				**0.088**	**0.031**	**0.006**	0.01	0.087	0.905
D2				**0.189**	**0.033**	**0**	0.156	0.091	0.087
PA*D1							0.019	0.02	0.338
PA*D2							0.008	0.021	0.7

Table S11
Hierarchical regression analysis results with sample size estimates as dependent variable and power analysis (PA), condition and their interaction as predictors.

	Model 1			Model 2			Model 3		
	Coef	(SE)	p	Coef	(SE)	p	Coef	(SE)	p
Small									
ΔR^2	0.004		0.354	0.009		0.393	0.03		0.039*
PA	8.736	9.411	0.354	8.957	9.426	0.343	-30.364	18.286	0.098
D1				-8.02	36.745	0.827	-200.873	100.407	0.047
D2				40.135	38.588	0.299	-200.629	105.021	0.057
PA*D1							47.686	23.353	0.042
PA*D2							60.006	24.489	0.015
Medium									
ΔR^2	0.007		0.211	**0.033**		**0.029**	0.001		0.873
PA	-5.732	4.564	0.211	**-5.348**	**4.515**	**0.238**	-8.329	8.89	0.35
D1				**8.061**	**17.6**	**0.647**	-1.808	48.814	0.97
D2				**46.033**	**18.483**	**0.014**	21.81	51.057	0.67
PA*D1							2.397	11.353	0.833
PA*D2							6.082	11.906	0.61
Large									
ΔR^2	0.053		0.001	**0.047**		**0.005**	0.011		0.285
PA	-13.076	3.81	0.001	**-12.672**	**3.737**	**0.001**	-6.302	7.318	0.39

DOI: 10.1177/0956797616647519

D1				**9.29**	**14.567**	**0.524**	25.464	40.184	0.527
D2				**47.466**	**15.297**	**0.002**	105.38	42.03	0.013
PA*D1							-3.863	9.346	0.68
PA*D2							-14.577	9.801	0.138

Table S12

Hierarchical regression analysis results with power estimates as dependent variable and statistical knowledge (SK), condition and their interaction as predictors.

	Model 1			Model 2			Model 3		
	Coef	(SE)	p	Coef	(SE)	p	Coef	(SE)	p
Small									
ΔR^2	0		0.75	**0.176**		**0**	0.002		0.821
SK	-0.003	0.01	0.75	**-0.002**	**0.01**	**0.851**	-0.001	0.016	0.965
D1				**0.067**	**0.032**	**0.038**	0.036	0.153	0.815
D2				**0.221**	**0.034**	**0**	0.289	0.165	0.081
SK*D1							0.005	0.022	0.833
SK*D2							-0.01	0.024	0.672
Medium									
ΔR^2	0.007		0.229	**0.251**		**0**	0.013		0.159
SK	0.011	0.009	0.229	**0.013**	**0.008**	**0.11**	-0.007	0.014	0.626
D1				**0.101**	**0.028**	**0**	-0.067	0.132	0.612
D2				**0.246**	**0.029**	**0**	-0.013	0.142	0.926
SK*D1							0.025	0.019	0.195
SK*D2							0.039	0.021	0.063
Large									
ΔR^2	0.019		0.043	**0.132**		**0**	0.017		0.122
SK	0.02	0.01	0.043	**0.022**	**0.009**	**0.021**	-0.004	0.015	0.799
D1				**0.086**	**0.031**	**0.007**	-0.182	0.147	0.219
D2				**0.188**	**0.033**	**0**	-0.072	0.159	0.652
SK*D1							0.04	0.022	0.065
SK*D2							0.039	0.023	0.097

Table S13

Hierarchical regression analysis results with sample size estimates as dependent variable and statistical knowledge (SK), condition and their interaction as predictors.

	Model 1			Model 2			Model 3		
	Coef	(SE)	p	Coef	(SE)	p	Coef	(SE)	p
Small									
ΔR^2	0.011		0.126	0.009		0.386	0.015		0.205
SK	16.597	10.792	0.126	16.816	10.799	0.121	-7.785	18.032	0.666
D1				-8.365	36.585	0.819	-305.541	172.555	0.078
D2				40.122	38.426	0.298	-158.955	185.634	0.393
SK*D1							44.559	25.3	0.08
SK*D2							29.78	27.323	0.277
Medium									
ΔR^2	0		0.851	**0.034**		**0.027**	0.012		0.263
SK	-0.994	5.271	0.851	**-0.682**	**5.208**	**0.896**	-11.921	8.707	0.172
D1				**8.939**	**17.645**	**0.613**	-119.156	83.321	0.154
D2				**46.934**	**18.533**	**0.012**	-54.234	89.636	0.546
SK*D1							19.2	12.217	0.118
SK*D2							15.152	13.193	0.252
Large									

DOI: 10.1177/0956797616647519

ΔR^2	0.005		0.308	**0.05**		**0.005**	0.001		0.946
SK	-4.592	4.494	0.308	**-4.259**	**4.403**	**0.334**	-5.973	7.406	0.421
D1				**11.181**	**14.915**	**0.454**	-11.91	70.868	0.867
D2				**49.35**	**15.666**	**0.002**	38.667	76.239	0.613
SK*D1							3.464	10.391	0.739
SK*D2							1.592	11.222	0.887
